# Supplementary figures and images for: A molecular phylogenetic appraisal of the acanthostomines Acanthostomum and Timoniella and their position within Cryptogonimidae (Trematoda: Opisthorchioidea)
Source: PeerJ. 2017 Dec 11;5:e4158. doi: 10.7717/peerj.4158 (PMC5729820; doi:10.7717/peerj.4158)

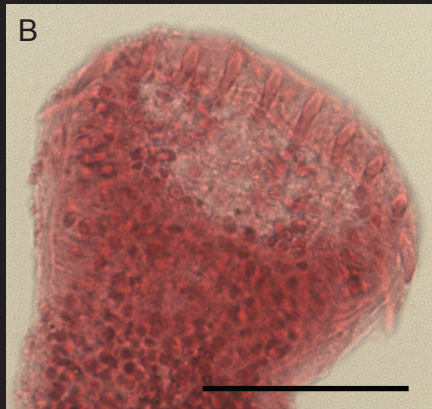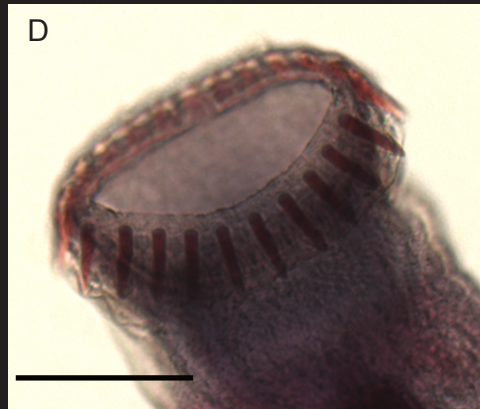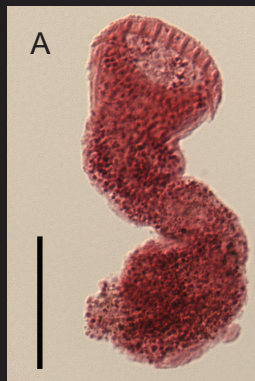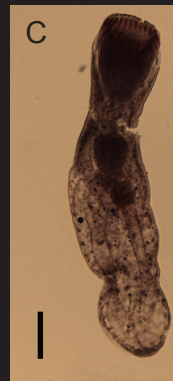

Supplement: Figure S1 — A. cf. americanum: (A) Ventral view, scale bar 100 µm; (B) Detail of the anterior end showing the rows of spines, scale bar 50 µm. T. cf. loossi: (C) Ventral view, scale bar 100 µm; (D) Detail of the anterior end showing the rows of spines, scale bar 100 µm. [file peerj-05-4158-s001.pdf]

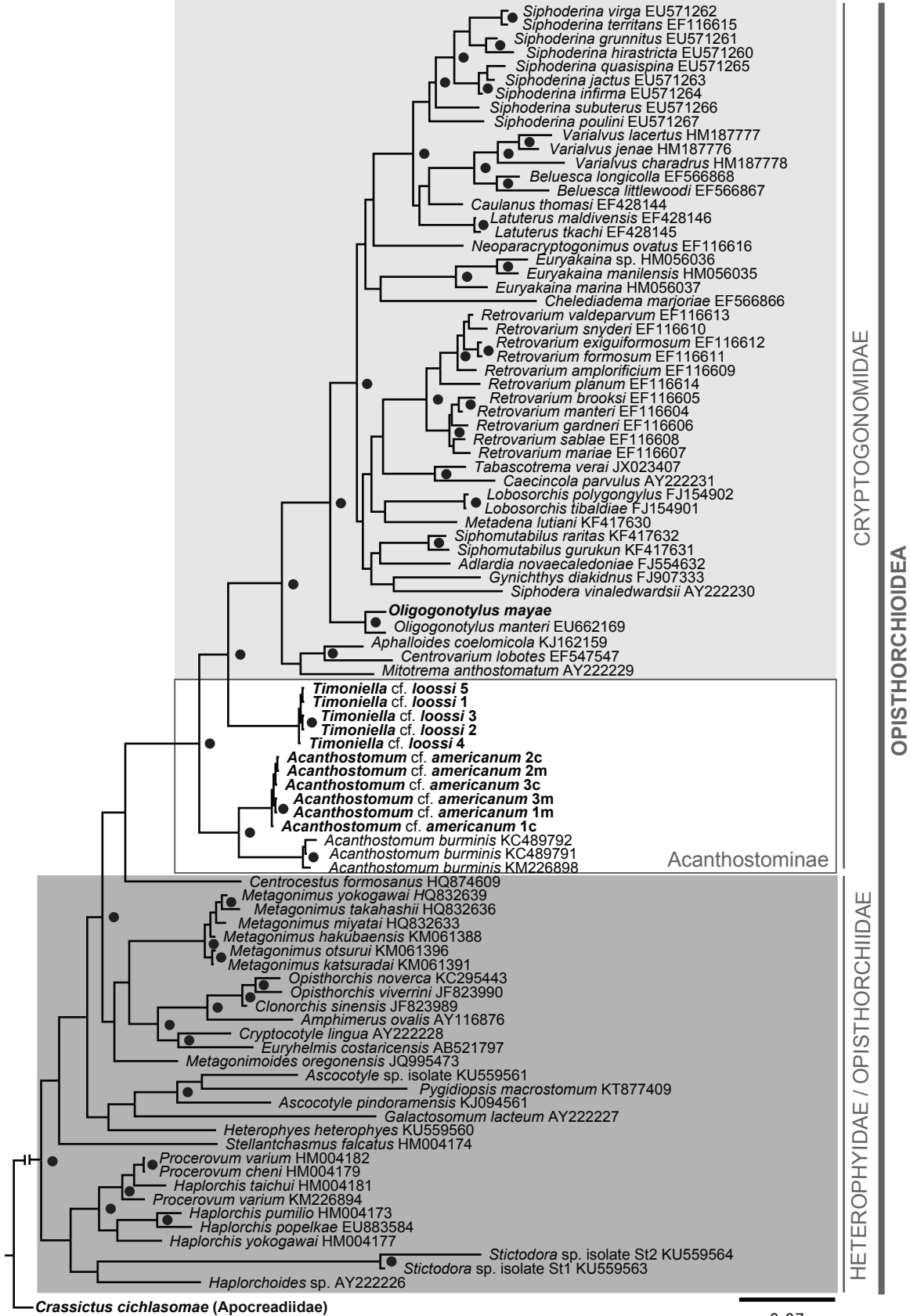

Supplement: Figure S2 — The scale bar represents the number of nucleotide substitutions per site. Codes following taxon names are cross-referenced in Table 1. Filled circles above/below branches represent Bayesian posterior probability ≥ 0.95. [file peerj-05-4158-s002.pdf]

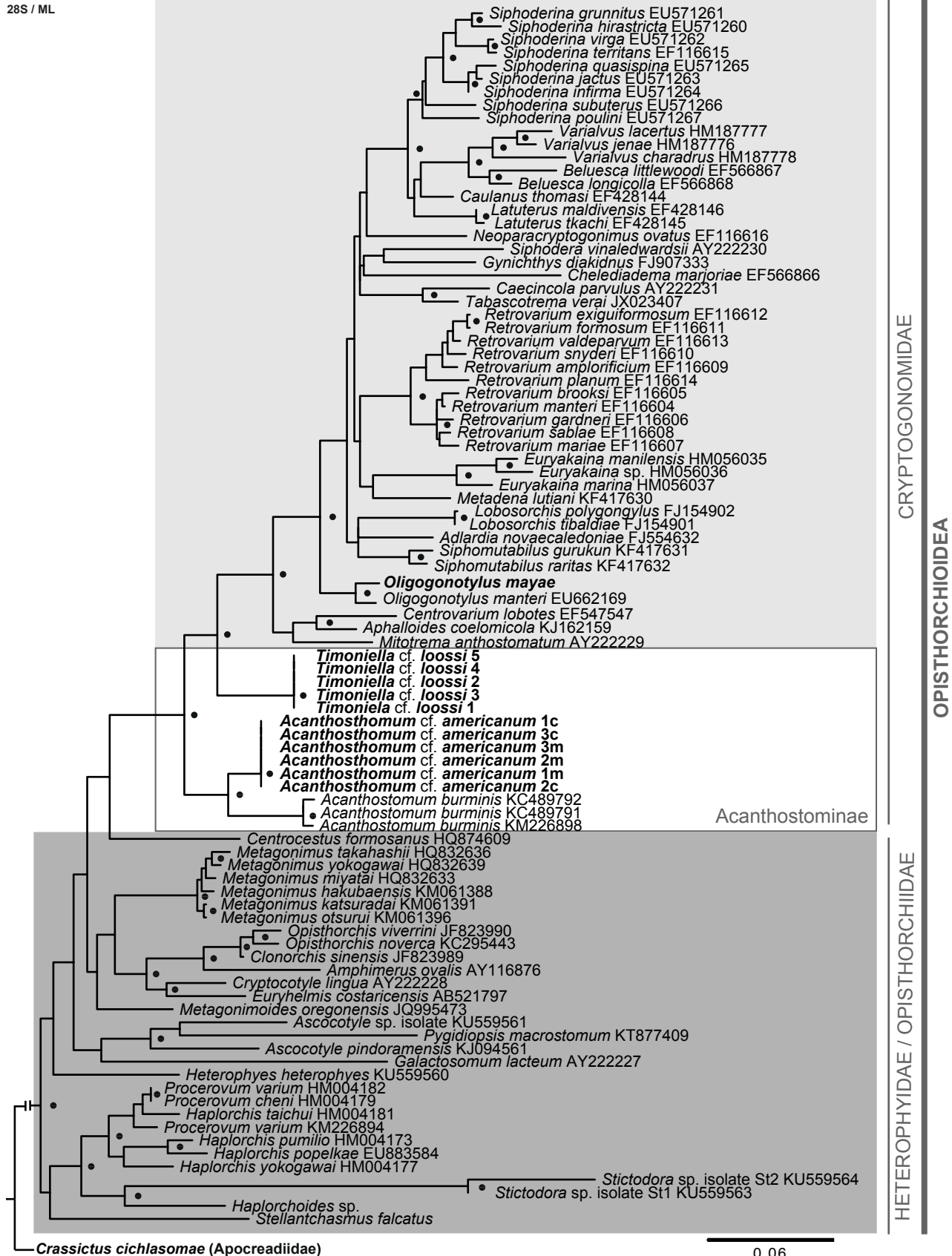

Supplement: Figure S3 — The scale bar represents the number of nucleotide substitutions per site. Codes following taxon names are cross-referenced in Table 1. Filled circles above/below branches represent bootstrap values ≥ 75%. [file peerj-05-4158-s003.pdf]

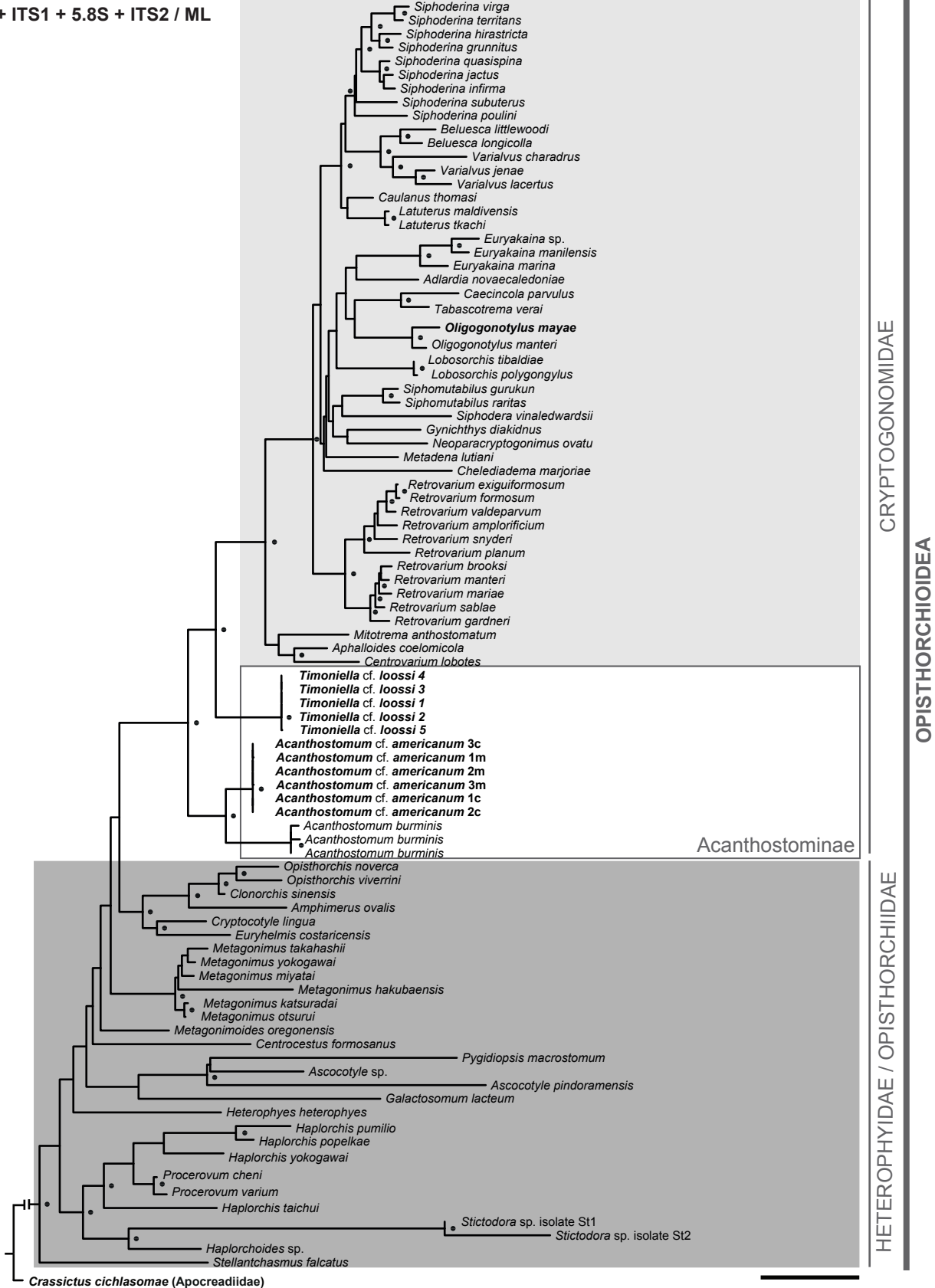

Supplement: Figure S4 — The scale bar represents the number of nucleotide substitutions per site. Codes following taxon names are cross-referenced in Table 1. Filled circles above/below branches represent bootstrap values ≥ 75%. [file peerj-05-4158-s004.pdf]
