# Supplementary material for: A molecular phylogenetic appraisal of the acanthostomines Acanthostomum and Timoniella and their position within Cryptogonimidae (Trematoda: Opisthorchioidea)
Source: PeerJ. 2017 Dec 11;5:e4158. doi: 10.7717/peerj.4158 (PMC5729820; doi:10.7717/peerj.4158)
Supplement: Table S1 [file peerj-05-4158-s005.pdf]

Table S1. List of analysed taxa for 28S and ITS1 + 5.8S + ITS with their GenBank accession numbers (new sequences in bold fonts).

Taxa	28S	ITS1	5.8S	ITS2
***Acanthostomum* cf. *americanum* 1m**	**MG383499**	**MG383512**	**MG383512**	**MG383512**
***Acanthostomum* cf. *americanum* 2m**	**MG383500**	**MG383513**	**MG383513**	**MG383513**
***Acanthostomum* cf. *americanum* 3m**	**MG383501**	**MG383514**	**MG383514**	**MG383514**
***Acanthostomum* cf. *americanum* 1c**	**MG383496**	**MG383509**	**MG383509**	**MG383509**
***Acanthostomum* cf. *americanum* 2c**	**MG383497**	**MG383510**	**MG383510**	**MG383510**
***Acanthostomum* cf. *americanum* 3c**	**MG383498**	**MG383511**	**MG383511**	**MG383511**
Acanthostomum burminis	KM226898	-	-	-
Acanthostomum burminis	KC489791	-	-	-
Acanthostomum burminis	KC489792	-	-	-
Adlardia novaecaledoniae	FJ554632	-	-	-
Amphimerus ovalis	AY116876	-	-	-
Aphalloides coelomicola	KJ162159	-	-	-
Ascocotyle pindoramensis	KJ094561	KJ094560	-	-
Ascocotyle sp.	KU559561	-	KU674957	KU674957
Beluesca littlewoodi	EF566867	EF566870	EF566870	EF566870
Beluesca longicolla	EF566868	EF566871	EF566871	EF566871
Caecincola parvulus	AY222231	-	-	-
Caulanus thomasi	EF428144	EF428141	EF428141	EF428141
Centrocestus formosanus	HQ874609	-	KJ630836	KJ630836
Centrovarium sp.	EF547547	-	-	-
Chelediadema marjoriae	EF566866	EF566869	EF566869	EF566869
Clonorchis sinensis	JF823989	JQ048576	JQ048576	JQ048576
Crassicutis cichlasomae	**MG383508**	**MG383521**	**MG383521**	**MG383521**
Cryptocotyle lingua	AY222228	KJ641523	KJ641523	KJ641523
Cryptogonimidae gen	HM056036	HM056034	HM056034	HM056034
Euryakaina manilensis	HM056035	-	-	-
Euryakaina marina	HM056037	-	-	-
Euryhelmis costaricensis	AB521797	AB521797	AB521797	AB521797
Galactosomum lacteum	AY222227	-	-	-
Gynichthys diakidnus	FJ907333	FJ907332	FJ907332	FJ907332
Haplorchis popelkae	EU883584	-	EU883584	EU883584
Haplorchis pumilio	HM004173	AY245706	AY245706	AY245706

Table S1. List of analysed taxa for 28S and ITS1 + 5.8S + ITS with their GenBank accession numbers (new sequences in bold fonts).

Taxa	28S	ITS1	5.8S	ITS2
Haplorchis taichui	HM004181	AY245705	AY245705	AY245705
Haplorchis yokogawai	HM004177	-	-	AB517590
Haplorchoides sp.	AY222226	-	-	-
Heterophyes heterophyes	KU559560	-	KU674951	KU674951
Latuterus maldivensis	EF428146	EF428143	EF428143	EF428143
Latuterus tkachi	EF428145	EF428142	EF428142	EF428142
Lobosorchis polygongylus	FJ154902	FJ154900	FJ154900	FJ154900
Lobosorchis tibaldiae	FJ154901	FJ154899	FJ154899	FJ154899
Metadena lutiani	KF417630	KF417627	KF417627	KF417627
Metagonimoides oregonensis	JQ995473	-	-	-
Metagonimus hakubaensis	KM061388	-	KM061397	KM061397
Metagonimus katsuradai	KM061391	-	KM061400	KM061400
Metagonimus miyatai	HQ832633	-	HQ832615	HQ832615
Metagonimus otsurui	KM061396	-	KM061403	KM061403
Metagonimus takahashii	HQ832636	-	HQ832618	HQ832618
Metagonimus yokogawai	HQ832639	KJ631740	KJ631740	KJ631740
Mitotrema anthostomatum	AY222229	-	-	-
Neoparacryptogonimus ovatus	EF116616	-	EF116631	EF116631
Oligogonotylus mayae	**MG383507**	**MG383520**	**MG383520**	**MG383520**
Oligogonotylus manteri	EU662169	EU662191	EU662191	EU662191
Opisthorchis noverca	KC295443	-	KC109193	KC109193
Opisthorchis viverrini	JF823990	EU038141	AY584735	AY584735
Procerovum cheni	HM004179	-	HM004164	HM004164
Procerovum varium	KM226894	-	HM004167	HM004167
Pygidiopsis macrostomum	KT877409	-	KT877410	KT877410
Retrovarium amplorificium	EF116609	EF116623	EF116635	EF116635
Retrovarium brooksi	EF116605	EF116629	EF116641	EF116641
Retrovarium exiguiformosum	EF116612	EF116626	EF116638	EF116638
Retrovarium formosum	EF116611	EF116625	EF116637	EF116637
Retrovarium gardneri	EF116606	-	EF116640	EF116640
Retrovarium manteri	EF116604	EF116621	EF116633	EF116633
Retrovarium mariae	EF116607	EF116618	EF116630	EF116630

Table S1. List of analysed taxa for 28S and ITS1 + 5.8S + ITS with their GenBank accession numbers (new sequences in bold fonts).

Taxa	28S	ITS1	5.8S	ITS2
Retrovarium planum	EF116614	EF116622	EF116634	EF116634
Retrovarium sablae	EF116608	EF116617	EF116642	EF116642
Retrovarium snyderi	EF116610	EF116624	EF116636	EF116636
Retrovarium valdeparvum	EF116613	EF116627	EF116639	EF116639
Siphodera vinaledwardsii	AY222230	-	-	-
Siphoderina grunnitus	EU571261	EU571257	EU571257	EU571257
Siphoderina hirastricta	EU571260	EU571255	EU571255	EU571255
Siphoderina infirma	EU571264	EU571256	EU571256	EU571256
Siphoderina jactus	EU571263	EU571253	EU571253	EU571253
Siphoderina poulini	EU571267	EU571254	EU571254	EU571254
Siphoderina quasi sp.ina	EU571265	EU571259	EU571259	EU571259
Siphoderina subuterus	EU571266	-	-	-
Siphoderina territans	EF116615	EF116620	EF116632	EF116632
Siphoderina virga	EU571262	EU571258	EU571258	EU571258
Siphomutabilus gurukun	KF417631	KF417628	KF417628	KF417628
Siphomutabilus raritas	KF417632	KF417629	KF417629	KF417629
Stellantchasmus falcatus	HM004174	-	KJ630833	KJ630833
Stictodora sp. isolate St1	KU559563	-	-	-
Stictodora sp. isolate St2	KU559564	-	-	-
Tabascotrema verai	JX023407	JX023379	JX023379	JX023379
***Timoniella* cf. *loosi* 1**	**MG383502**	**MG383515**	**MG383515**	**MG383515**
***Timoniella* cf. *loosi* 2**	**MG383503**	**MG383516**	**MG383516**	**MG383516**
***Timoniella* cf. *loosi* 3**	**MG383504**	**MG383517**	**MG383517**	**MG383517**
***Timoniella* cf. *loosi* 4**	**MG383505**	**MG383518**	**MG383518**	**MG383518**
***Timoniella* cf. *loosi* 5**	**MG383506**	**MG383519**	**MG383519**	**MG383519**
Varialvus charadrus	HM187778	HM187781	HM187781	HM187781
Varialvus jenae	HM187776	HM187779	HM187779	HM187779
Varialvus lacertus	HM187777	HM187780	HM187780	HM187780
